# A Role for Behavior in the Relationships Between Depression and Hostility and Cardiovascular Disease Incidence, Mortality, and All-Cause Mortality: the Prime Study

**DOI:** 10.1007/s12160-016-9784-x

**Published:** 2016-03-15

**Authors:** K. M. Appleton, J. V. Woodside, D. Arveiler, B. Haas, P. Amouyel, M. Montaye, J. Ferrieres, J. B. Ruidavets, J. W. G. Yarnell, F. Kee, A. Evans, A. Bingham, P. Ducimetiere, C. C. Patterson, for the PRIME study group

**Affiliations:** Department of Psychology, Bournemouth University, Poole, BH12 5BB UK; School of Medicine, Dentistry and Biomedical Sciences, Queen’s University Belfast, Belfast, BT12 6BJ UK; Department of Epidemiology and Public Health, University of Strasbourg, EA3430 Strasbourg, France; The Lille Monica Project, INSERM U744, Lille, France; The Toulouse MONICA Project, INSERM UMR1027, Toulouse, France; The Coordinating Center, INSERM U780, Hôpital Paul Brousse, Villejuif, France

**Keywords:** Depression, Hostility, Behavior, Cardiovascular disease, Mortality, Social support

## Abstract

**Background:**

Behavioral factors are important in disease incidence and mortality and may explain associations between mortality and various psychological traits.

**Purpose:**

These analyses investigated the impact of behavioral factors on the associations between depression, hostility and cardiovascular disease(CVD) incidence, CVD mortality, and all-cause mortality.

**Methods:**

Data from the PRIME Study (*N* = 6953 men) were analyzed using Cox proportional hazards models, following adjustment for demographic and biological CVD risk factors, and other psychological traits, including social support.

**Results:**

Following initial adjustment, both depression and hostility were significantly associated with both mortality outcomes (smallest SHR = 1.24, *p* < 0.001). Following adjustment for behavioral factors, all relationships were attenuated both when accounting for and not accounting for other psychological variables. Associations with all-cause mortality remained significant (smallest SHR = 1.14, *p* = 0.04). Of the behaviors included, the most significant contribution to outcomes was found for smoking, but a role was also found for fruit and vegetable intakes and high alcohol consumption.

**Conclusions:**

These findings demonstrate well-known associations between depression, hostility, and mortality and suggest the potential importance of behaviors in explaining these relationships.

## Introduction

Various negative psychological traits have previously been associated with cardiovascular disease (CVD) incidence, CVD mortality, and all-cause mortality. Early studies reported positive associations between type A behavior and CVD incidence and mortality (e.g., 1, 2), and studies since have reported positive associations between hostility and CVD [3–6], hostility and all-cause mortality [6], and anger expression and CVD [3, 6, 7]. Depression has been repeatedly positively associated with CVD and all-cause mortality (e.g., 4, 8–12), and studies have demonstrated various associations between CVD and a variety of other psychological traits (4, 6, 13 and see 14 for a review).

Explanations for associations between psychological traits, CVD, and mortality are largely based in physiology. Negative psychological traits and CVD are most commonly linked via increases in autonomic nervous system (ANS) responding to stress or ‘cardiac reactivity’ (increases in heart rate, systolic, and diastolic blood pressure in response to stress), and via increased activity of the hypothalamic-pituitary-adrenal (HPA) axis, with subsequent impacts on immune and inflammatory systems [4, 9, 10, 15–17], although other pathways may also play a role [4, 17]. In relation to increased ANS responding, Pizzi et al., 2008 [4], found differences in heart rate variability between depressed and non-depressed individuals, type A behaviors, hostility, anger, and aggression have all been associated with increased cardiac reactivity [14, 18], and individuals defined as hostile or angry compared to others have demonstrated increased heart rate and increased blood pressure in response to stressful tasks [19, 20]. In relation to the HPA axis, Pope & Smith, 1991 [21], reported increased cortisol levels in hostile compared to low-hostile men during everyday activities, Suarez et al., 1998 [22], showed increased cortisol in hostile men under stress compared to low-hostile men, and Steptoe et al., 2000 [23], found increased free cortisol in angry individuals compared to non-angry individuals when under high job strain. In relation to immune and inflammatory activity, Pizzi et al., 2008 [17], found differences in the levels of various inflammatory markers between depressed and non-depressed individuals, Howren et al., 2009 [16], found positive associations between depression and various inflammatory markers in a meta-analysis, and Stewart et al., 2008 [24], found associations between depression, hostility, and inflammatory markers, in combination. Empana et al., 2005 [11], also found positive associations between depressive symptoms and inflammatory markers after adjustment for classic CVD risk factors. Cardiac reactivity, increased cortisol and increased inflammatory responses have also been demonstrated in association with CVD and mortality [4, 11, 17, 25, 26].

Behavioral explanations, however, are also possible. Various negative psychological traits and CVD are associated with poor health behaviors, such as smoking, poor diet, and low physical activity [3, 6, 27], and it may be these poor health behaviors that account for the relationships between negative psychological traits and CVD incidence and mortality. DiMatteo et al., 2000 [28], demonstrate associations between depression and anxiety and poor treatment compliance. Scherwitz et al., 1992 [29] report increased smoking, marijuana use, alcohol, and energy intake in hostile compared to low-hostile individuals. Shin et al., 2005 [30], find a strong association between anger and poor sleep, Falk Dahl & Dahl, 2010 [13], report associations between anxiety, alcohol problems, and low levels of exercise, and Siegler et al., 1992 [31], demonstrate positive associations between hostility and smoking, BMI, lipid levels, and caffeine intake 21–23 years later. In relation to disease and mortality, previous analyses of the cohort under consideration have shown associations between smoking and CVD events and all-cause mortality, after controlling for various demographic variables [32], associations between alcohol intake patterns and CVD, after controlling for demographic and classic CVD risk factors [33], and a contribution from smoking, fruit and vegetable intakes, physical activity, and alcohol intakes to the observed socioeconomic gradient in CVD events and all-cause mortality after controlling for demographic and classic CVD risk factors [34].

Associations between psychological traits and disease outcome, however, are complicated by possible overlap between psychological variables, and possible interactions between psychological variables and behavior [3, 6, 7, 19].

Particular emphasis has also previously been given to the conciliatory role of the more positive psychological variable of social support. Social support is well-recognized to have positive health benefits [35, 36]. In relation specifically to CVD, emotionally supportive social support has been found to protect against CVD [4], and low levels of emotional support have been associated with negative health outcomes [4, 37]. Rosengren and colleagues [37], for example, reported a protective effect of social support for new CVD events in middle-aged men, and Krumholz and colleagues [38], showed emotional support as a strong predictor of cardiac events in hospital patients when accounting for disease severity on hospital admission and instrumental help for assisting living. Social support has also been found to result in reduced ANS reactivity, reduced cortisol reactivity in response to stressors, increased inflammatory and immune responding [36, 39–42], and has been linked to CVD and mortality via these mechanisms. Effects of stress on cardiac function have been found to be lower in those with social support compared to those without [35], and social isolation in combination with high stress has been found to predict mortality in post Myocardial Infarction (MI) patients [43].

Interactions with negative psychological traits, however, may limit the impact of social support in certain individuals [20, 44]. Of relevance to the preceding discussions, depression, hostility, and anger particularly, may undermine the possible beneficial influence of social support [44]. Depression is often characterized by social withdrawal and a negative interpretation of the actions of others [28]. Hostility and anger are defined by mistrust and cynicism of others, a negative interpretation of others, and often also a negative attitude or reaction to others [6, 20]. Social withdrawal, negative interpretations of others, including authority, and negative attitudes and behaviors towards others will limit the possibility of and possible benefit attained from social support by depressed, hostile, or angry individuals [6, 28, 39]. Holt-Lunstad and colleagues [20], for example, demonstrate decreased ratings of the friendliness of friends from hostile compared to non-hostile individuals despite no differences in observed friend behaviors. Lepore [39] also demonstrated that hostile individuals do not benefit from the support of strangers, while non-hostile individuals do benefit.

Associations between psychological traits and CVD have been found not only in relation to CVD incidence but also for CVD prognosis and progression [3, 4, 9]. Impact on the relationships between negative psychological traits and CVD by behavioral variables could provide valuable suggestions for intervention, both for the prevention of CVD and for its treatment [3, 6]. This analysis aimed to investigate the impact of behavioral variables on the associations between negative psychological traits and CVD incidence, CVD mortality, and mortality from all-causes. Four lifestyle behaviors were considered—smoking, alcohol intake, fruit and vegetable consumption, and physical activity. Analyses were conducted both with and without prior accounting for other psychological variables, including a measure of social support.

## Methods

### The PRIME Study

Analyses were conducted on the data from the Prospective Epidemiological Study of Myocardial Infarction (PRIME) study, in which data on various psychological traits, on various lifestyle behaviors, and on CVD incidence and mortality are available over a 10-year follow-up period for 9709 men, aged 50–59 years, from France and Northern Ireland who were free of cardiovascular disease (coronary heart disease or cerebrovascular disease) at study entry. Full details of the PRIME study are provided elsewhere [45].

### Psychological Traits

Psychological traits were assessed at the start of the study using a 70 item questionnaire, derived from a number of validated questionnaires, including the Framingham Type A scale [1], the Cook-Medley Hostility scale [46], the Welsh Pure Depression Scale [47], and the MONICA scales for the assessment of social interactions [48], plus additional questions derived by researchers based on the current literature at the time [49]. Sixty-nine items from this questionnaire were subsequently analyzed by principal component analysis (with varimax rotation) (one item was optional and, due to low response rates, was excluded from the analysis). A composite questionnaire and principal component analysis was used to avoid the use of multiple similar questionnaire items from different validated questionnaires, due to the extensive assessment schedule for the whole study for participants. The principal component analysis revealed eight factors, explaining 37 % of the variance, but inspection of individual factor loadings and composite factors, and reference to an earlier analysis of the same dataset [49] resulted in a decision to limit the analysis outcomes to five factors, explaining 29 % of the variance. These five factors utilized 58 items from the questionnaire. All items with a factor loading of less than 0.30 on any factor were ignored. Based on their component questions, factors were labelled depression (16 items), competitiveness (14 items), hostility (10 items), social support (8 items), and anger/impatience (10 items). Cronbach’s alpha for reliability was high for scales labelled depression (α = 0.71), hostility (α = 0.80), and social support (α = 0.81) but was very low (even after the removal of individual items) for scales labelled competitiveness (highest α = 0.26) and anger (highest α = 0.16). Due to their low reliability, these two scales were not used further for analyses. Analyses were thus conducted for two negative psychological traits—depression and hostility, with consideration for social support (as below). All questions were answered using a variety of response formats, but these were subsequently re-scaled to result in a score per question of between 0 and 1. Scores for each scale were created per person by adding scores for all relevant items and dividing by the number of items, to result in a score for each scale per individual between 0 and 1, where higher scores denote stronger feeling.

### Lifestyle Behaviors

Four lifestyle behaviors were assessed by self-report questionnaire, also completed at the start of the study. Fruit and vegetable intakes were assessed using a food frequency questionnaire of various fruits and vegetables, and responses were defined in terms of number of portions of fruit, fruit juice, and vegetables consumed per day. Responses to physical activity questions based on the amount of time undertaken: ‘*sitting or standing still*/*walking*/*lifting or carrying moderately heavy objects* (*5*–*10 kg*) *or doing activities of similar effort*/*lifting or carrying very heavy objects* (*more than 10 kg*) *or doing activities of similar effort*, *on an average day at work*’; ‘*walking*/*cycling to and from work*’; ‘*walking*’; ‘*playing sports or doing exercise*’; were converted into metabolic equivalent scores/week. Responses to questions on smoking (‘*Have you ever smoked*?’; ‘*What do you smoke*?’; ‘*How many do you smoke on an average day*?’) were subsequently divided into five categories, based on commonly used categories: never smoked; ex-smoker; and tertiles: smoking less than 15 cigarettes per day; smoking 15–20 cigarettes per day; smoking more than 20 cigarettes per day. Alcohol intake was assessed using a week-based dietary recall requesting number of measures consumed of various alcoholic beverages. Responses were subsequently divided into five categories, based on an abstainers group and four drinking groups defined by quartiles of consumption: none; 1–128 ml/week; 129–265 ml/week; 266–461 ml/week; and 462 or more ml/week.

### CVD Incidence, Mortality, and All-cause Mortality

Cardiovascular disease incidence, mortality from cardiovascular disease and mortality from all causes were assessed for a 10-year period from the start of the study via medical records. Cardiovascular events included validated myocardial infarction and/or stroke (fatal and non-fatal). All reported cases were validated by an independent medical committee comprising a medical investigator from each PRIME centre and three independent cardiologists. A separate committee was established with an independent neurologist to validate the stroke events [32, 50]. Death certificates were obtained for all men who died and causes of death classified using the International Classification of Diseases (ICD) ninth revision [50].

### Analyses

The Cox proportional hazards models were used to investigate associations between depression, hostility, and all outcome variables. In model 1, relationships were described after adjusting for ten demographic and biological risk factors associated with CVD incidence or mortality: age, marital status (married/single), country of residence (NI/France), socio-economic status (low/medium/high, based on “material conditions”— a composite variable based on home ownership and the number of cars, baths/showers and toilets [32]), systolic blood pressure (mmHg), cholesterol (mg/l), HDL cholesterol (mg/l), height (m), BMI (kg/m^2^), and diabetes (present/absent). In model 2, relationships were described after adjusting also for four behavioral factors: fruit and vegetable intake, physical activity, current smoking, and alcohol intake. In model 3, relationships were described after adjusting for demographic and biological risk factors, and scores on the other negative psychological trait (hostility or depression) and social support. In model 4, relationships were described after adjusting for demographic and biological risk factors, for other psychological traits and for the four behavioral factors. Measures of fruit and vegetable intake and physical activity were square root transformed prior to analyses, due to their skewed distributions. Measures of smoking and alcohol were included in analyses along with their interactions with country in light of previous findings of differing effects of smoking and alcohol use on our outcomes in Northern Ireland and France [32]. Measures of depression, hostility, and social support were standardized to have a mean of zero and a standard deviation of one prior to all analyses, to allow easier interpretation of effect sizes. Results are presented as standardized hazard ratios—the hazard rate multiplier associated with a one standard deviation change in the psychological variable holding other variables in the model constant. Differences in the relationships between depression/hostility score and each outcome variable between models 1 and 2, and between models 3 and 4 demonstrate the impact of the four lifestyle behaviors on these associations. In models 3 and 4, differences in the relationships, demonstrate the impact of the behavioral variables after taking account of the impacts of other psychological variables. While associations between psychological traits and behaviors are well-recognized, independent associations between behaviors and CVD outcomes are also possible. Analyses were conducted only on participants who were free from CVD at the start of the study, and who provided data for all psychological measures. All analyses were of time to first outcome event. For CVD incidence (fatal or non-fatal), we used the time to first manifestation (i.e., if a man had a CVD incident and then died some time afterwards from a second CVD event then the first CVD event was the defining event for CVD incidence, but for CVD mortality and all-cause mortality, the second CVD event was the defining one). Follow-up finished on all men at the time of death. Censoring of CVD incidence (fatal or non-fatal) and of CVD mortality occurred at the time of any non-CV death. The proportional hazards assumption was checked by including interactions between covariates and follow-up time in the models as time-dependent covariates.

## Results

Data from 6953 men, who provided complete data sets, were available for analysis. Of these, 317 suffered a CVD event (fatal or non-fatal) over the 10-year follow-up, 56 men died from CVD, and 354 men died from all causes. Descriptive statistics for the sample are provided in Table 1.

**Table 1:** Descriptive statistics for the sample (*N* = 6953)

	Mean	St. dev.	Minimum	Maximum
Age (years)	54.8	2.9	48	64
Systolic blood pressure (mmHg)	133.6	18.8	79	226
Cholesterol (mg/DL)	2.22	0.38	0.79	6.15
HDL cholesterol (mg/DL)	0.49	0.13	0.10	1.50
Height (m)	1.73	0.07	1.43	2.00
BMI (kg/m^**2**^)	26.5	3.3	15.8	47.6
Depression score (0–1)	0.22	0.18	0	1
Hostility score (0–1)	0.48	0.29	0	1
Fruit and vegetable intakes (portions of fruit, fruit juice and vegetables/day)	2.6	1.4	0	21
Physical activity (metabolic equivalent scores/week)	95	64	0	422
Country of residence (N (%))	France—5001 (72 %); Northern Ireland - 1952 (28 %)			
Smoking (N (%) per category)	Never smoked—2151 (31 %); Ex-smoker—2977 (43 %); Currently smoking less than 15 cigarettes per day—1000 (14 %); Currently smoking 15–20 cigarettes per day—539 (8 %); Currently smoking more than 20 cigarettes per day—286 (4 %);			
Alcohol (N (%) per category)	None—1175 (17 %); 1–128 ml/week—1522 (22 %); 129–265 ml/week—1511 (22 %); 266–461 ml/week—1356 (19 %); 462 or more ml/week—1389 (20 %).			
Diabetes (N (%) present)	200 (3 %)			
Disease or mortality (number of cases)	CVD incidence—317; CVD mortality—56; all-cause mortality—354.			

### Depression

Scores for depression ranged from 0 to 1, mean = 0.22 (SD = 0.18). Depression scores were significantly higher in France compared to Northern Ireland (t(6951) = 2.01, *p* = 0.05), in single compared to married/cohabiting individuals (t(6950) = 5.82, *p* < 0.01) and in individuals with diabetes compared to those without (t(6951) = 3.85, *p* < 0.01). Weak, but statistically significant correlation coefficients were found between depression scores and hostility (*r* = 0.26, *p* < 0.01) and social support (*r* = −0.21. *p* < 0.01) scores.

Standardized hazards ratios, confidence intervals and significance for the associations between depression scores, CVD incidence, CVD mortality, and mortality from all causes are displayed in Table 2. Following adjustment for demographic and biological risk factors (model 1), depression was associated with both mortality outcomes. Following additional adjustment for behavioral variables (model 2), effect sizes reduced slightly for all relationships, and only the relationship with mortality from all causes remained significant. Where hostility and social support were adjusted for alongside demographic and biological risk factors (model 3), only the relationship between depression score and mortality from all causes was significant. Following additional adjustment for the behavioral variables (model 4), effect sizes reduced slightly for all outcomes, but the relationship with all-cause mortality remained significant.

**Table 2:** Standardized hazards ratios, confidence intervals and statistical significance for the association between depression scores, CVD incidence, CVD mortality and mortality from all-causes

	Model 1—adjusted for demographic and biological^a^ risk factors			Model 2—adjusted for demographic and biological^a^ and behavioral^b^ risk factors			Model 3—adjusted for demographic and biological^a^ and other psychological^c^ risk factors			Model 4—adjusted for demographic and biological^a^, other psychological^c^ and behavioral^b^ risk factors		
	SHR	95 % CI	Sig.	SHR	95 % CI	Sig.	SHR	95 % CI	Sig.	SHR	95 % CI	Sig.
CVD incidence (*N* = 317)	1.10	0.99, 1.22	0.08	1.07	0.96, 1.19	0.23	1.09	0.97, 1.22	0.14	1.06	0.95, 1.19	0.28
CVD mortality (*N* = 56)	**1.28**	**1.02**, **1.62**	**0.03**	1.25	0.99, 1.57	0.06	1.22	0.96, 1.55	0.11	1.20	0.94, 1.52	0.15
All-cause mortality (*n* = 354)	**1.20**	**1.09**, **1.32**	<**0.001**	**1.16**	**1.05**, **1.27**	<**0.01**	**1.15**	**1.04**, **1.27**	<**0.01**	**1.12**	**1.02**, **1.24**	**0.02**

^a^Demographic and biological risk factors—age, marital status, country of residence, socio-economic status, systolic blood pressure, cholesterol, HDL cholesterol, height, BMI, and diabetes

^b^Behavioral risk factors—fruit and vegetable intake, physical activity, current smoking, and current alcohol consumption

^c^Other psychological risk factors—hostility score and social support score

SHR—Standardized hazard ratio (per 1 standard deviation increase in depression score)

Significant relationships (*p* < 0.05) are emboldened

### Hostility

Scores for hostility ranged from 0 to 1, mean = 0.48 (SD = 0.29). Hostility scores were significantly higher in France compared to Northern Ireland (t(6951) = 27.42, *p* < 0.01), and in individuals with diabetes compared to those without (t(6951) = 4.71, *p* < 0.01). Hostility scores were also weakly correlated with depression (*r* = 0.26, *p* < 0.01) and social support (*r* = −0.32, *p* < 0.01) scores.

Standardized hazards ratios, confidence intervals, and significance for the associations between hostility scores, CVD incidence, CVD mortality, and all-cause mortality are displayed in Table 3. Following adjustment for demographic and biological risk factors (model 1), hostility was associated with both mortality outcomes. Following additional adjustment for behavioral variables (model 2), effect sizes reduced for all relationships, and only the relationship with CVD mortality remained significant. Where depression and social support were adjusted for alongside demographic and biological risk factors (model 3), relationships between hostility score and both mortality outcomes were significant. Following additional adjustment for the behavioral variables (model 4), effect sizes reduced slightly for all outcomes, and the relationship with CVD mortality became non-significant, while the relationship with all-cause mortality remained significant. 

Of the behavioral variables, fruit and vegetable intake was negatively associated with CVD incidence. Smoking was positively associated with CVD incidence and, at the highest level with all-cause mortality. Alcohol intake at the highest level was also associated with all-cause mortality (data not shown).

**Table 3:** Standardized hazards ratios, confidence intervals and statistical significance for the association between hostility scores, CVD incidence, CVD mortality, and mortality from all-causes

	Model 1—adjusted for demographic and biological^a^ risk factors			Model 2—adjusted for demographic and biological^a^ and behavioral^b^ risk factors			Model 3—adjusted for demographic and biological^a^ and other psychological^c^ risk factors			Model 4—adjusted for demographic and biological^a^, other psychological^c^ and behavioral^b^ risk factors		
	SHR	95 % CI	Sig.	SHR	95 % CI	Sig.	SHR	95 % CI	Sig.	SHR	95 % CI	Sig.
CVD incidence (*N* = 317)	1.11	0.98, 1.25	0.09	1.08	0.96, 1.22	0.21	1.10	0.97, 1.25	0.13	1.09	0.96, 1.24	0.20
CVD mortality (*N* = 56)	**1.43**	**1.06**, **1.93**	**0.02**	**1.37**	**1.02**, **1.86**	**0.04**	**1.38**	**1.01**, **1.90**	**0.05**	1.33	0.97, 1.83	0.08
All-cause mortality (*n* = 354)	**1.24**	**1.10**, **1.38**	<**0.001**	**1.18**	**1.05**, **1.33**	<**0.01**	**1.18**	**1.05**, **1.34**	<**0.01**	**1.14**	**1.01**, **1.29**	**0.04**

^a^Demographic and biological risk factors—age, marital status, country of residence, socio-economic status, systolic blood pressure, cholesterol, HDL cholesterol, height, BMI, and diabetes

^b^Behavioral risk factors—fruit and vegetable intake, physical activity, current smoking, and current alcohol consumption

^c^Other psychological risk factors—depression score and social support score

SHR—Standardized hazard ratio (per 1 standard deviation increase in hostility score)

Significant relationships (*p* < 0.05) are emboldened

## Discussion

These findings firstly demonstrate relationships between both depression and hostility and CVD mortality and all-cause mortality, when adjusting for demographic and biological risk factors. These relationships have previously been demonstrated elsewhere (e.g., 3, 9, 12).

Secondly, these relationships were attenuated by the inclusion of lifestyle behaviors in the predictive models. These findings demonstrate the importance of behavioral variables in these relationships. Behaviors have previously been hypothesized as the mechanism through which various characteristics and traits such as depression and hostility affect disease and mortality. Whooley and colleagues [51], for example, find no association between depression and CVD events after controlling for alcohol use, smoking, physical activity, and medication non-adherence. Chida & Steptoe [3] find no association between hostility and anger and CVD incidence after controlling for smoking, physical activity, and BMI as well as socio-economic status. Chida & Hamer [15] also found a reduction in associations with cardiac reactivity after controlling for behavioral variables. A role for behavior may have considerable implications for treatment and secondary prevention. Behaviors may offer an alternative route for intervention than that offered through the treatment of psychological variables. Repeated research shows beneficial impacts of behavioral interventions (e.g., 52).

Effects sizes are small, but these effect sizes represent only the direct effects of the considered lifestyle behaviors on the relationships between depression, hostility, and all outcome variables. Additional effects are also likely, as a result of impacts of the lifestyle behaviors on demographic and biological risk factors and on the psychological variables themselves. Fruit and vegetable intake and physical activity, for example, are known to impact on many of the biological risk factors for CVD, including some of those controlled in analyses here—blood pressure, cholesterol levels, BMI, and diabetes (e.g., 53–57). Smoking and alcohol use can also have impacts on these biological risk factors (e.g., 58, 59). The lifestyle behaviors investigated may also impact on depression, hostility, and social support (e.g., 60–62). The distinction between behavioral, biological, and psychological risk factors is often unclear, and relationships between all three are likely to be much more complex than is suggested by our analyses. The lifestyle factors investigated furthermore may only represent a subset of those behaviors that impact on the relationships between psychological health and disease/mortality (e.g., 28–31).

Of the behaviors investigated, smoking contributed most significantly to mortality, although evidence for a role from fruit and vegetable intake and high alcohol consumption was also found. The important contribution of smoking to CVD disease and mortality is well-recognized (e.g., 32). Associations between fruit and vegetable intake and CVD disease, mortality, and all-cause mortality are also well-known (e.g., 63, 64), and associations between alcohol intake and all-cause mortality are well-recognized (e.g., [65]). Of interest in our data, physical activity was not important for CVD incidence or mortality, but this result may be specific to the measurement of physical activity used, and our specific sample characteristics—middle-aged men.

The lifestyle behaviors were found to be important, furthermore, both when other psychological variables were also accounted for and when not. Comparisons between models 1 and 3 suggest that the additional psychological variables do impact on the primary relationships with all outcomes, although effect sizes are smaller than those for the lifestyle behaviors. These small effects may have resulted from the measures used but are likely to also demonstrate the high inter-relation between psychological variables [43]. The similar patterns in findings for depression and hostility also suggest a close relationship between these variables. Associations between psychological variables have previously been reported [3, 6, 7, 19, 24].

Interestingly, none of the relationships were attenuated entirely by the inclusion of the lifestyle behaviors in analyses, and relationships between both depression and hostility and all-cause mortality remained significant. Independent associations between depression, hostility, and all-cause mortality have previously also been found elsewhere. Miller and colleagues [6], for example, also report a significant independent association between hostility and CVD incidence and mortality after controlling for behavioral variables. These authors, however, also suggest that accounting for behaviors in studies may not always be adequate [6]. Behaviors in addition to those frequently measured may have additional impact. In relation to depression and hostility, medication compliance [28], and quality as opposed to quantity of social support [44] are obvious suggestions, but other characteristics and behaviors, such as those related to childhood experiences or exposures, may also have impacts in these relationships [3]. These early experiences are, however, very difficult to control for.

The strengths of these analyses clearly lie in the prospective nature of the data on which the analyses are conducted, the large sample size and the long (10 year) follow-up period involved. Limitations lie in the measures used for the assessment of the psychological variables, the limited behaviors that were measured, the self-report measures used to study these behaviors, and the possibility that these behaviors may have changed over the course of the study period—assessments of behavior were only made at the start of the study. Psychological variables were not assessed using complete validated measures [66], but were instead assessed using a composite questionnaire. We have no data to compare our questionnaire scores to the scores of validated questionnaires, but similar levels of depressive symptoms have been reported using the CES-D scale in older (13.2 %) and elderly (21.5 %) general populations from Europe [67], and repeat analyses using the questions from the Welsh Pure Depression Scale and the questions from the Cook-Medley Hostility scale in place of our composite measures of depression and hostility reveal the same patterns as those presented (data not shown). It is possible that our findings are a result at least in part of our use of a composite measure, but given the comparability between our findings and those of others, we think this is unlikely. The limitations of self-report for our behavioral measures and our use of limited behaviors are acknowledged, but health behaviors are known to typically cluster highly, thus assessment of further behaviors may have little impact on our findings. The assessment of behavior only at the start of the study however, not only limits our abilities to monitor changes over time but also limits our abilities to study these using more formal mediation analyses due to the temporal precedence requirement for mediation models [68]. While novel procedures are currently under development (e.g., see 69), formal mediation analyses for use in survival (time-to-event) data are currently not well established [70]. The analysis was also restricted to middle aged (50–59 year old) men, while sex and age differences in CVD, mortality, heath behaviors, negative psychological traits, and social support are well-known [3, 9, 17, 23, 24, 29, 41].

In conclusion, this analysis demonstrates positive associations between depression and hostility and mortality from CVD and all causes. These associations, however, were reduced when accounting for lifestyle behaviors. These findings demonstrate the importance of health behaviors in the relationships between negative psychological traits and mortality. These findings may suggest possibilities for treatment and secondary prevention.
